# Platelet Membrane Receptor Proteolysis: Implications for Platelet Function

**DOI:** 10.3389/fcvm.2020.608391

**Published:** 2021-01-08

**Authors:** Jiayu Wu, Johan W. M. Heemskerk, Constance C. F. M. J. Baaten

**Affiliations:** ^1^Department of Biochemistry, Cardiovascular Research Institute Maastricht, Maastricht University, Maastricht, Netherlands; ^2^Institute for Molecular Cardiovascular Research (IMCAR), University Hospital Rheinisch-Westfälische Technische Hochschule (RWTH) Aachen, Aachen, Germany

**Keywords:** ADAM, calpain, caspase, coagulation factors, MMP, platelets, receptor proteolysis

## Abstract

The activities of adhesion and signaling receptors in platelets are controlled by several mechanisms. An important way of regulation is provided by proteolytic cleavage of several of these receptors, leading to either a gain or a loss of platelet function. The proteases involved are of different origins and types: (i) present as precursor in plasma, (ii) secreted into the plasma by activated platelets or other blood cells, or (iii) intracellularly activated and cleaving cytosolic receptor domains. We provide a comprehensive overview of the proteases acting on the platelet membrane. We describe how these are activated, which are their target proteins, and how their proteolytic activity modulates platelet functions. The review focuses on coagulation-related proteases, plasmin, matrix metalloproteinases, ADAM(TS) isoforms, cathepsins, caspases, and calpains. We also describe how the proteolytic activities are determined by different platelet populations in a thrombus and conversely how proteolysis contributes to the formation of such populations.

## Introduction

According to a common concept of thrombosis and hemostasis, damage or injury of a vessel wall and ensuing exposure of extracellular matrix components to the blood stream triggers platelets from the circulation to become adherent and to assemble into a thrombus, thus limiting the extravasation of blood (1, 2). Within a thrombus, however, distinct types of activated platelets can be recognized, exhibiting different functions, although partial overlap between the populations exists (3).

The process of thrombus formation is considered to be initiated by von Willebrand factor (VWF) binding to exposed collagen or laminin in the damaged vessel wall, followed by shear-dependent platelet binding to VWF through the glycoprotein (GP)Ib-V–IX complex (1, 3, 4). The flow-dependent adhesion of platelets to those and other extracellular matrix components is stabilized by a panel of integrins, including integrin α_2_β_1_, α_IIb_β_3_, and α_6_β_1_ (adhesive platelet population), while the initial activation of platelets is achieved by signaling via the collagen/laminin receptor, glycoprotein VI (GPVI). This provokes the release of thromboxane A_2_ and the secretion of granular contents including ADP and, via these autocrine agents, the subsequent recruitment of additional flowing platelets, which assemble into a growing thrombus via α_IIb_β_3_-fibrinogen interactions (aggregating platelet population) (1). Upon prolonged high intracellular rises in Ca^2+^, platelets develop a procoagulant phenotype that is characterized by the surface membrane exposure of phosphatidylserine and by the inactivation of integrin α_IIb_β_3_ (procoagulant platelet population) (5). Phosphatidylserine-exposing platelets, usually located around a thrombus, provide a negatively charged membrane surface, which supports coagulation factor binding and the formation of tenase and prothrombinase complexes (6, 7). Thrombin, which is generated at these phosphatidylserine sites, triggers the formation of fibrin fibers, which consolidate the platelet thrombus into a stable clot sealing the breach in a vessel wall (2). Independently of such activation processes, platelet heterogeneity can be achieved by aging and an accompanied inactivation (3). The latter changes are considered to have apoptosis-like properties (8).

Although these platelet activation processes are relatively well-studied as a function of the platelet environment, only since recently it is becoming clear that a multitude of proteases present in plasma or produced by platelets themselves are important for the distinct properties of platelet populations, often by cleaving specific receptors. Here, we review current knowledge how proteases act on platelet receptors and the platelet membrane surface. We describe how these are activated, their targets, their effect on platelet functions, and the consequences for platelet population formation. An overview of the discussed proteases, their known targets, and their effects on platelet function is given in Supplementary Table 1.

## Coagulation and Related Proteases

The coagulation process is accomplished by consecutive activity of several plasmatic serine proteases, which always circulate as zymogen precursors and, once activated, provide complex feedback loops to trigger other coagulation or anticoagulation factors (1, 9). Serine proteases (serine endopeptidases) are defined as proteolytic enzymes with a serine residue in their active site; they usually have a multidomain structure containing β-barrel regions and multiple surface loops surrounding the active site (10). Relevant coagulation serine proteases that have been reported to act on platelets are thrombin, factor Xa, and plasmin (11), as summarized in Supplementary Table 1. A structurally related serine protease is trypsin.

**Figure 1:**
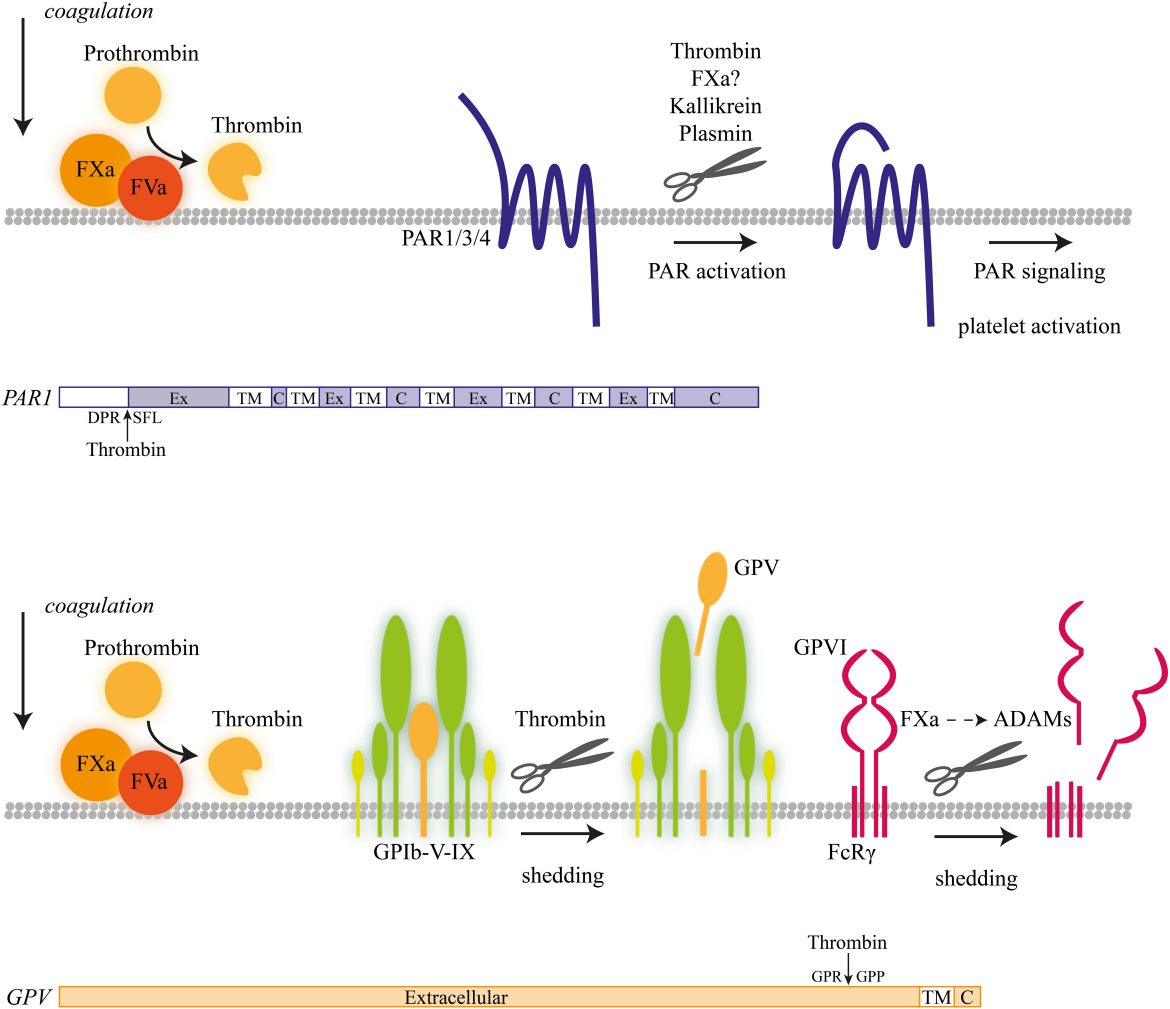
Coagulation-induced receptor proteolysis. Proteolytic activation mechanism of coagulation factors at the platelet membrane, progressing to thrombin-induced activation of proteolytic-activated receptor (PAR) isoforms and shedding of glycoprotein V (GPV) and GPVI with a schematic overview of proteolytic cleavage sites.

In the extrinsic coagulation process triggered by tissue factor, the protease factor Xa is produced from its zymogen factor X by factor VII/VIIa (1, 9). Cleavage into factor Xa is greatly enhanced in the presence of phosphatidylserine-exposing membranes, such as present on procoagulant platelets (12, 13). On such platelets, the so-called tenase complex, with as protease factor IXa and as cofactor VIIIa, catalyzes the conversion of factor X into factor Xa. Subsequently, the protease factor Xa along with cofactor Va (prothrombinase complex) catalyzes the conversion of prothrombin into α-thrombin (1, 14). Both factor Xa and thrombin cleave a range of substrates, including proteolytic-activated receptors (PARs), while thrombin also cleaves fibrinogen to form fibrin clots (Figure 1). A further substrate of thrombin is the serine protease plasmin, which is produced on endothelial cells from its precursor plasminogen, and acts as an essential factor in fibrinolysis (15). The activity of all three serine proteases—factor Xa, thrombin, plasmin—is tightly regulated by circulating serpins (serine protease inhibitors, such as antithrombin), which avidly bind to and inactivate the catalytic domains of the proteases (16). The binding of thrombin to endothelial-bound thrombomodulin, in addition, activates the anticoagulant pathway of protein C (17).

Other serine proteases such as trypsin (in the intestine) are also released as zymogens (trypsinogen) that require proteolytic cleavage (hetero- or autocatalytic) to give the active proteolytic enzyme. Trypsin (not present in blood plasma) has a strong preference for cleavage of the PAR2 receptors (18), although one report demonstrates trypsin IV-induced cleavage of PAR4 (19). Overall, it appears that the various serine proteases that affect platelets first need to be converted into an active form via proteolysis and that all are prone to inactivation by serpins. As a consequence, the platelet activation responses to these proteases are confined in space and time.

### Thrombin

α-Thrombin is known as a potent platelet agonist. On platelets, it carries out the activating potential by inducing proteolytic events and furthermore by binding to the GPIb-V–IX complex (1, 20–22). Established thrombin substrates include PAR receptors, coagulation factors V, VIII, and XIII, and fibrinogen (23).

In human platelets, α-thrombin cleaves the N-terminal extracellular site of isoforms PAR1 and PAR4 (24, 25). The proteolysis liberates a new N-terminus, which autocatalytically activates the receptor molecule (26, 27). By ensuing signaling via G-proteins (in particular Gqα) (24, 28), thrombin induces a wide range of platelet responses, including shape change, Ca^2+^ fluxes, integrin activation, and secretion (23).

α-Thrombin easily activates and cleaves the PAR1 receptor (Arg^41^-Ser^42^) with an EC_50_ of around 0.1 nM, due to the presence of a second thrombin binding site on this receptor. The hexapeptide SFLLRN (TRAP6) acts as a specific agonist of PAR1, as it represents the neo-N-terminal site of the cleaved receptor. In addition, the α-thrombin precursor form meizothrombin (μ-thrombin), recognized as an obligatory intermediate during prothrombin conversion, contains proteolytic activity toward the platelet receptor but at a fairly reduced rate (29, 30). For the activation and cleavage of PAR4, higher thrombin concentrations are required, in the order of 5 nM. Hence, it is considered that PAR1 cleavage by thrombin precedes PAR4 cleavage (23).

While human platelets express only very low copy numbers of PAR3, this isoform is more abundant on rodent platelets, where it functionally replaces the PAR1 isoform. Thus, on human platelets, PAR3 cleavage hardly contributes to the thrombin-induced responses (23). The other receptor family member, PAR2, is not known as a thrombin receptor, but rather as a cleavable receptor for the coagulation factors VIIa and Xa (31, 32). On platelets, it is lacking.

Another high-affinity receptor for thrombin is the GPIbα chain of the GPIb-V–IX complex (33). Upon thrombin-induced platelet activation, GPIbα is considered to cooperate with the two PAR receptors (34). However, thrombin binding can also facilitate another, this time proteolytic, effect on the GPIb-V–IX complex, namely by cleaving the extracellular domain of GPV (35). Interestingly, mice lacking GPV showed an increased platelet activation and arterial thrombosis formation for reasons that are not well-understood (36). The cleaved, soluble GPV fragment has been used as a marker of platelet activation *in vivo* (37).

Next to binding to platelet receptors proteins, thrombin carries out additional proteolytic events at the platelet surface. On activated, procoagulant platelets, thrombin cleaves coagulation factor V (whether or not platelet-derived) into factor Va, thus enhancing the prothrombinase activity (38). Furthermore, thrombin cleaves platelet-bound fibrinogen to produce fibrin polymers that form the shield of so-called coated platelets (39, 40). The latter process preferentially occurs on procoagulant platelets, secondary to processes like ballooning and phosphatidylserine exposure (39).

A poorly understood finding is that platelet activation with the TRAP6 peptide, similarly to α-thrombin but at a lower extent, can induce cleavage or PAR1 (Arg^41^-Ser^42^) in a manner antagonized by serpin but not by metalloproteinase inhibition (41). This may imply the presence of another trypsin-like protease implicated in TRAP6-induced platelet activation.

### Factor Xa and Other Coagulation Proteases

Factor Xa itself is generated (from non-cleaved factor X) at the surface of procoagulant, phosphatidylserine-exposing platelets, where it serves to produce thrombin (42, 43). The combined activities of factor Xa and thrombin can be measured in thrombin generation assays under stasis and are also detectable under flow conditions in whole blood assays (12, 44). Intriguingly, the factor Xa receptor PAR2, present on endothelial cells (45), does not play a significant role in human platelet activation (46). While for endothelial cells a receptor cross-talk has been reported of factor Xa mediating PAR1 cleavage (47), this is not known for platelets or other PAR1-overexpressing cells (23). A recent report stipulates that in plasma- and blood-based systems, the blockage of factor Xa with rivaroxaban inhibits a factor Xa-driven platelet activation pathway via PAR1 (48). However, it cannot be ruled out that, in this setting, thrombin itself is the receptor-cleaving protease.

Another not well-understood effect of factor Xa on platelets is its reported ability to mediate coagulation-dependent shedding of the extracellular domain of GPVI (49). Evidence for this effect came from the finding that this shedding of GPVI was blocked by factor Xa inhibition (rivaroxaban), rather than by thrombin inhibition (dabigatran, hirudin). Markedly, the receptor shedding is also sensitive to metalloproteinase inhibition, which can point to an indirect rather than a direct role of the factor Xa enzyme (50).

Another plasma protease that is proposed to modulate PAR1 cleavage is kallikrein. It has been reported that a kallikrein preparation via proteolysis can disarm or inhibit PAR1 signaling (51). A different proposal is that kallikrein first binds to integrin α_IIb_β_3_ and then potentiates ADP-induced platelet activation through prior cleavage of PAR1 (52). The evidence for this mechanism so far is pharmacological.

Recombinant factor VIIa (NovoSeven) is incidentally used to treat patients with coagulation of platelet defects (53), for instance in patients with Glanzmann's thrombasthenia (54). The mechanism of factor VII's proteolytic activity is peculiar, since the idea is that factor VIIa (in the apparent absence of tissue factor) enhances the generation of factor Xa and thrombin at the platelet surface and thereby promotes thrombin-induced thrombus formation under flow (55, 56). Factor VIIa hence can play a role in the serine protease cascade at the platelet membrane.

The receptor chain GPIbα, due to its large and charged extracellular domain and its abundance on platelets, has been considered as a “sink” of several coagulation factors, i.e., next to (pro)thrombin, also factors VII(a), VIII, and XI (11). Binding of factor XI to GPIbα was identified as a proteolytic feedback pathway for thrombin generation in the setting of angiotensin-II-induced hypertension (57). Earlier reports on GPIbα-dependent factor XI binding and activation on platelets (58) were later partly modified by proposing that also the platelet ApoER2 receptors act as factor XI ligands (59). During the intrinsic coagulation pathway, factor XIa cleaves factor IX, but so far, no proteolytic events of factor IXa or XIa on platelet membrane proteins have been reported.

### Plasmin and Cathepsins

To become an active serine protease, plasmin needs to be cleaved from its precursor plasminogen by the enzymes tissue or urokinase plasminogen activator. Several reports indicate that this proteolytic cleavage is promoted at the surface of activated (phosphatidylserine-exposing) platelets. One proposal is that polyphosphates released from platelets allow binding of factor XII(a) on platelets, which, in some way, enhances plasminogen cleavage into plasmin (60, 61). Plasmin is a key enzyme in the fibrinolytic process, causing proteolytic fibrin degradation, but in addition, plasmin is known to act on platelets. Reports indicate that plasmin cleaves and activates the receptors PAR1 and/or PAR4, however at a distinct site as thrombin, thereby suppressing thrombin-induced platelet activation (62, 63). Plasmin inactivation is accomplished by serpins such as the plasmin activator inhibitor-I (PAI-1).

Concerning cathepsins, another class of plasma proteases secreted by leukocytes and other cells, only little is known of proteolytic effects on platelets. Most of the cathepsins act as cysteine proteases. It is described that the neutrophil-derived cathepsin G evokes platelet activation via the proteolytic cleavage of PAR4 (64). Similarly, the mesenchymal-cell-derived cathepsin K can activate the PAR4 receptor (65). On the other hand, platelets produce a broad-range inhibitor of cathepsins, called cystatin A, which was proposed to interfere in thrombus formation in a matrix metalloproteinase (MMP)-dependent manner; the proteolytic events here are still unresolved (66).

## Matrix Metalloproteinases

MMPs belong to the metzincin superfamily of zinc-dependent metalloproteinases. In the human genome, 23 different members of MMPs have been identified. Conventionally, these are grouped into the following classes: collagenases, gelatinases, stromelysins, matrilysins, and membrane-bound MMPs. The classification is based on the enzymes' substrates, the structural domains, and their pericellular localization (67). As an overall structure, MMPs are composed of a propeptide sequence, a catalytic domain, a hinge region, and a hemopexin domain. Many of the MMPs are secreted from cells, after which they remain at the cell membrane to carry out proteolytic functions (68).

In the cell, MMPs are produced as inactive zymogens (pro-MMPs), which require proteolytic removal of a propeptide sequence to become active. A result of this activation step is exposure of the enzyme's catalytic domain containing a Zn^2+^ ion (67). However, protection against undesired activity is still provided by a range of abundantly expressed tissue inhibitors of metalloproteinases (TIMPs), which avidly bind to the MMP catalytic domains. With respect to platelets, specific MMPs preferentially interact with TIMP isoforms, although there is a certain degree of redundancy. Other than the name “matrix metalloproteinase” suggests, most MMPs cleave not only extracellular matrix components but also other pericellular proteins (67), several of which regulate platelet functionality.

Of the 23 known MMPs, expression in human megakaryocytes and platelets on the transcript and/or protein level is confirmed for 13 isoforms (i.e., MMP1, 2, 3, 7, 9, 11, 12, 14, 15, 17, 19, 24, 25) (69, 70). In addition, all four TIMP isoforms are expressed, and these appear to be secreted along with the MMPs upon platelet activation (70–72). How the 13 expressed MMP isoforms contribute to the overall high MMP activity of (activated) platelets and to platelet function is still mostly unclear. At present, a role in platelets has been elucidated for the isoforms MMP1, 2, 9, 12, 13, and 14 (Supplementary Table 1).

### MMP1

MMP1 belongs to the collagenase class of MMPs and has a granular distribution in resting platelets (73). The latent pro-MMP1 requires processing into active MMP1 upon secretion (74). Once at the plasma membrane, (pro)MMP1 was found to colocalize with integrins α_IIb_β_3_ and α_2_β_1_ (74, 75).

**Figure 2:**
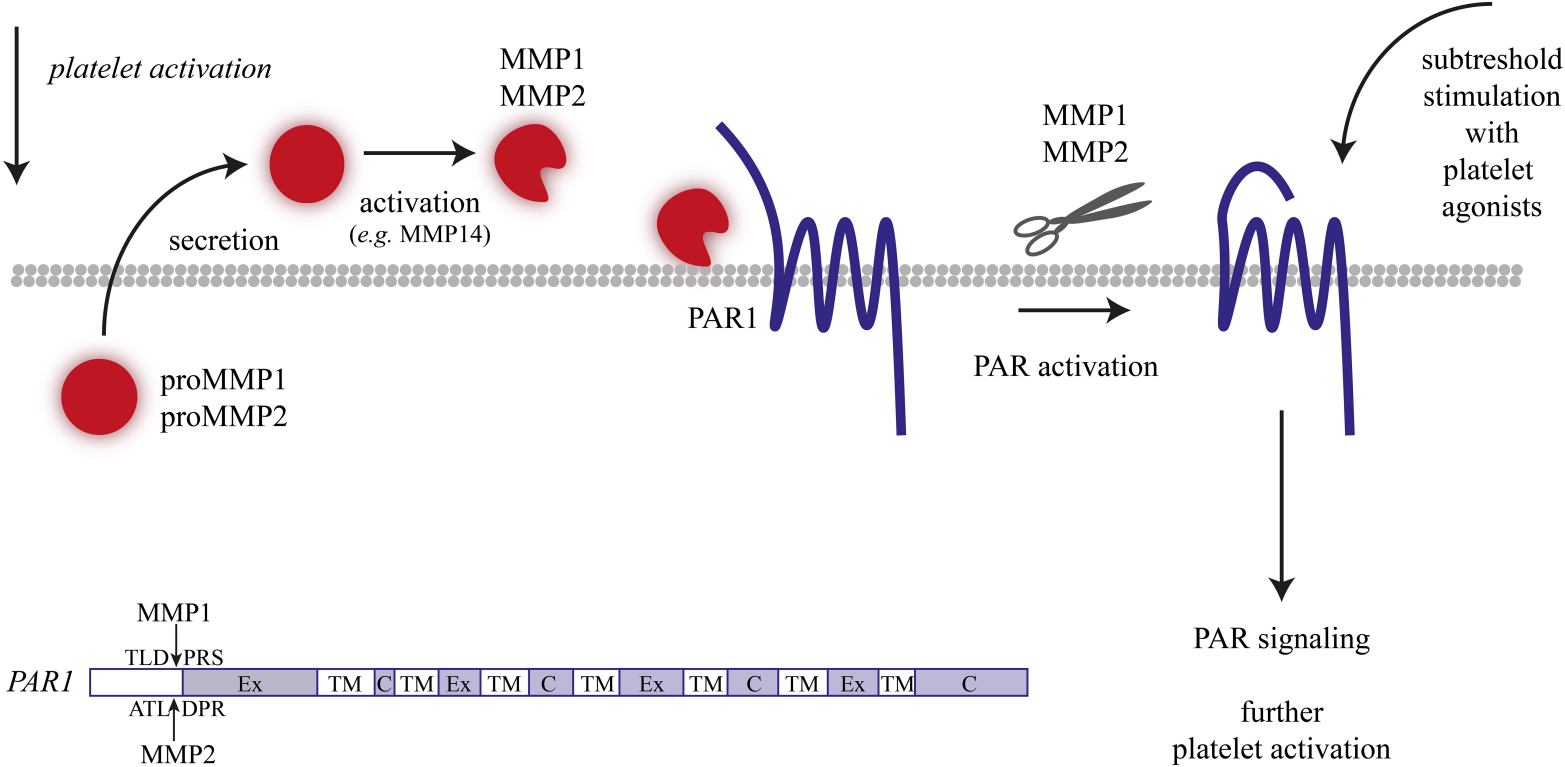
Matrix metalloproteinase (MMP)-1/2 induced platelet activation. Overview of mechanisms resulting in MMP-1/2 induced or enhanced platelet activation.

Active MMP1 is recognized as one of the several proteases that can cleave the N-terminal domain of the thrombin receptor, PAR1; the resulting fragment can activate PAR1 and trigger downstream signaling events (75). In agreement with the role of MMP1 in platelet activation, it was shown in two different *in vivo* thrombosis models using guinea pigs that inhibition of MMP1 suppressed intravascular thrombus formation and prolonged the time to vessel occlusion (75). Here, coadministration of a PAR1 antagonist did not increase the effects of MMP1 inhibition, thus suggesting overlap of the roles of MMP1 and PAR1. Similarly, in a human whole blood model of thrombus formation, the inhibition of MMP1 activity led to a reduction in platelet adhesion, aggregate formation, and P-selectin expression (73). Conversely, the treatment of human platelets with recombinant MMP1 potentiated thrombin-induced aggregation (74). While these findings collectively point to a functional role of MMP1 in cleaving the PAR1 receptors (Figure 2), the metalloproteinase may also stimulate and cleave other proteins on platelets.

### MMP2

As a member of the gelatinase class, MMP2 is the most extensively studied MMP isoform in the context of platelet function. During platelet activation with collagen or thrombin, pro-MMP2 is released into the plasma and then translocates to the platelet surface (76, 77), where it binds to integrin α_IIb_β_3_ (78). *In vivo*, the liberation of MMP2 has been detected at sites of vascular injury, in a manner relying on the extent of platelet activation (79). Along the same line, platelet inhibition by nitric oxide or prostacyclin appeared to inhibit the MMP2 release (76). While MMP2 itself is not considered as a platelet agonist, it enhances the activation process with subthreshold levels of agonists, as evidenced by an increased Ca^2+^ mobilization (76, 80). Evidence for such an enhancing role of MMP2 on platelet activation and (arterial) thrombus formation came from the use of MMP2 inhibitors and from mice with an MMP2 deficiency (76, 81). How these enhancing effects rely on proteolysis is still a matter of debate.

Several targets of MMP2 proteolysis have been identified, including the surface glycoproteins CD40L and CLEC2, of which extracellular soluble fragments are circulating in the plasma (82–84). Furthermore, MMP2 has been implicated in PAR1 activation by cleaving this receptor at a non-canonical site (i.e., different from the thrombin and MMP1 cleavage site), with integrin α_IIb_β_3_ serving as a cofactor (Figure 2) (85). Cleavage generated a PAR1-activating peptide, which then stimulated signaling events via the G-proteins Gqα and G12/13α. However, the MMP2-generated peptide did not stimulate the Giα pathway, which was considered as an explanation why MMP2 did not elicit full platelet activation (85).

### MMP9

Whether or not the gelatinase MMP9 is expressed in platelets at relevant copy numbers is a matter of debate. Some research groups have detected measurable MMP9 activity in platelets (86, 87), while other groups could not confirm this finding (74, 88). Treatment of human platelets with recombinant MMP9 suppressed in a dose-dependent way thrombin- and collagen-induced aggregation, phosphoinositide breakdown, Ca^2+^ mobilization, and thromboxane A_2_ formation, which effects could be linked to cyclic guanosine monophosphate (cGMP) elevation and protein kinase C inactivation (86, 87). Similarly, platelet-inhibitory properties of (plasma-derived) MMP9 were seen in collagen-induced thrombus formation *ex vivo* (73). In addition, in mice, genetic deficiency of MMP9 resulted in the formation of larger thrombi under flow with higher levels of platelet activation in terms of P-selectin and phosphatidylserine exposure (73). If and how these effects rely on MMP9 proteolytic activity is unclear, and no definitive MMP9 targets are known so far. On the other hand, indirect evidence suggests a role of MMP9 in the extracellular cleavage of platelet CD40L (89).

### MMP12

Platelet-expressed MMP12 (a metalloelastase) has been reported to enhance collagen-induced aggregation and α-granule secretion but not aggregation by other agonists (90). Possibly linked to this finding, MMP12 can induce extracellular cleavage of the signaling receptor CAECAM1 at multiple sites, resulting in soluble fragments, of which one could reproduce the potentiating effect of MMP12 on collagen-induced activation (90). Other proteolytic activities of this protease on platelets are not reported.

### MMP13

There is limited evidence in the literature for a proteolytic role of the plasma-derived MMP13 (a collagenase) on platelet function (Supplementary Table 1). One group indicated that, at pathologically relevant concentrations, purified MMP13, added to whole blood, binds to integrin α_IIb_β_3_ and GPVI and as such impairs human platelet aggregation and thrombus formation under flow (91). A distinct mechanism that was proposed is that MMP13-induced cleavage of VWF liberates a VWF product that more strongly interacts with platelets and partly loses its ability to adhere to collagen (92). In addition, MMP13 can (partially) degrade collagen, which may also alter the process of thrombus formation.

### MMP14

The isoform MMP14 is a membrane-type protease that remains tethered to the plasma membrane of resting and activated platelets. Its general function is supposed to be the breakdown of extracellular matrix components. Interestingly, upon GPVI-induced activation, caps of MMP14 were observed at the platelet surface, possibly indicating concentration of MMP activity (73). The MMP14 isoform likely cleaves a variety of protein substrates including pro-MMP2 and pro-MMP13 (93), which in turn alters platelet properties. Such a multisubstrate characteristic of MMP14 may also explain why inhibition of this isoform was found to increase collagen-dependent platelet activation and thrombus formation (73).

## Adam and Adamts Proteases

### ADAM10 and ADAM17

The ADAM proteases, as an abbreviation for “a disintegrin and metalloprotease,” belong to the adamalysin group of the metzincin metalloproteinases superfamily. Several of the ADAM isoforms have a profound influence on cellular functions, such as cell adhesion, migration, intracellular signaling, matrix degradation, and other proteolytic processes (94). As its name suggests, the ADAM molecular structure resembles that of other MMPs. Most ADAM proteins are made up of eight functional domains: a signal peptide domain; a prodomain present in the inactive form; a catalytic domain; disintegrin, cysteine-rich, and epidermal growth factor (EGF)-like domains (except for ADAM10 and ADAM17); a transmembrane domain; and a cytoplasmic tail (94, 95). Given the transmembrane localization with catalytic domain expressed extracellularly, ADAM-induced proteolysis occurs outside the cell surface near the plasma membrane.

Of the multiple ADAM domains, the catalytic domain—also called metalloprotease domain—is best known. It contains a highly conserved zinc-dependent endopeptidase motif with three histidine residues and a methionine turn in the active-site helix (94). Of the 21 presumed functional ADAMs, so far, 13 have been found to induce shedding of extracellular target (receptor) proteins. The other ADAM isoforms lack the endopeptidase motif and may be involved in transcellular cross-talk and cell adhesion via the disintegrin domain (94, 95). The common but not only mechanism of ADAM activation is the removal of the prodomain, thus liberating the Zn^2+^-dependent catalytic site.

In platelets, the isoforms ADAM10 and ADAM17 (also called TACE) are identified as major receptor sheddases of this family (96). Both ADAM10 and ADAM17 are single-pass transmembrane proteins that lack the EGF-like domain, although they only share 30% amino acid sequence identity. The list of recognized substrates for ADAM10 and ADAM17 is longer than that for other ADAM members (94), due to more extensive research.

Studies with cultured cells indicated that the expression of an active ADAM form at the cell surface relies on vesicular transport, cleavage of the prodomain by a convertase, and release by TIMPs blocking the ADAM active site (94). It is considered that ADAM10 is primarily blocked by TIMP1 and ADAM17 by TIMP3 (94).

**Figure 3:**
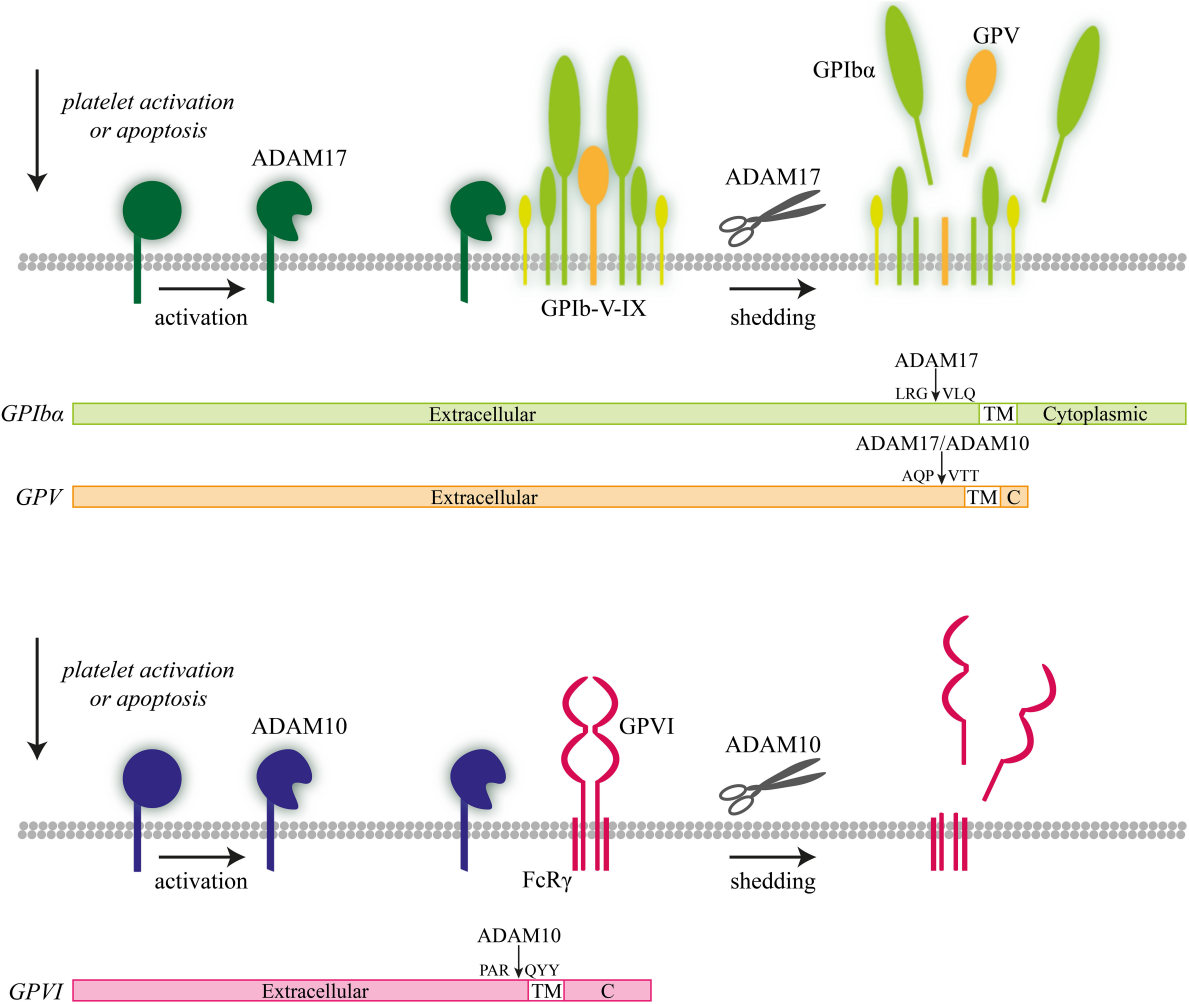
ADAM10- and ADAM 17-mediated proteolysis of platelet receptors. Illustration of ADAM10-mediated receptor shedding of glycoprotein V (GPV) and ADAM17-mediated receptor shedding of GPIbα with proteolytic cleavage sites indicated.

In platelets, the isoforms ADAM10 and ADAM17 appeared to have a partly overlapping target spectrum (Figure 3). *In vivo* and *in vitro* studies using deficient mice revealed that ADAM10 preferentially cleaves the extracellular domain of GPVI on activated platelets (97), whereas ADAM17 rather targets GPIbα (97–99). On the other hand, in the GPIb-V–IX complex, the GPV chain can be cleaved by both enzymes. Other platelet receptors targeted by the ADAM isoforms are CD84, Sema7A, Sema4D, and JAM-A (99–103). In all these cases, cleavage will lead to an altered interaction of platelets with their environment. A related finding was the ability of ADAMs to act as a sheddase of platelet-bound amyloid precursor protein (APP) (104).

While constitutively active ADAM10 is already present on resting platelets, ADAM activity was found to drastically increase upon platelet activation (105). The signaling and activation mechanisms that lead to ADAM10- and ADAM17-mediated receptor shedding are unraveled only partly. Literature suggests that both extracellular and intracellular signals can enhance the proteolytic activity of ADAM forms (Supplementary Table 1). Recognized ADAM-activating agents that induce receptor shedding of GPIbα and/or GPVI include (i) inhibitors of the oxidative phosphorylation like CCCP (97, 98); (ii) inhibitors of protein kinase A (106); (iii) inhibitors of calmodulin (97, 107); (iv) reactive oxygen species (108); and (v) the coagulation product fibrin (109). Recently, our group has shown that ADAM-mediated receptor cleavage is confined to highly activated platelets (thrombin/CRP-XL), with, as a result, a defined population of phosphatidylserine-exposing platelets showing low GPIbα and GPVI expression (110). A further set of signaling processes that can trigger receptor shedding includes activation of protein kinase C (97, 110), p38 mitogen-activated protein kinase (108), and apoptosis (110, 111). Recently, it was shown that the Bruton's tyrosine kinase (Btk) inhibitor ibrutinib induced a time- and dose-dependent shedding of the GPIb-IX complex by increasing ADAM17 activation (112). Platelets from leukemia patients treated with ibrutinib showed reduced expression of the GPIb-IX complex, and also in mice, ibrutinib treatment resulted in elevated levels of soluble GPIbα (112).

In terms of function, it is assumed that ADAM10- and ADAM17-mediated receptor cleavage protects the platelets from a continued activation, by limiting receptor activity and the downstream signaling pathways (113). Alternatively, it can be stated that shedding of adhesive receptors such as GPIbα may steer removal from the circulation. However, in mice, platelet ADAM17 (TACE) deficiency reduced the levels of soluble GPIbα fragments in the plasma without affecting platelet count (98). Curiously, a decreased platelet ADAM10 level has been suggested as an early indicator for Alzheimer's disease. This relates to the fact that ADAM10, as an α-secretase, induces non-amyloidogenic proteolysis of the amyloid precursor protein, thus suppressing formation of the pathologic Aβ form (104, 114).

### ADAMTS13

The isoforms of a disintegrin and metalloprotease with thrombospondin type 1 repeats (ADAMTS) are structurally highly similar to the ADAM proteases. While the enzymes of both families have in common a prodomain, catalytic, disintegrin, and cysteine-rich domains, the ADAMTS isoforms in addition contain one or more thrombospondin domains and a variable additional C-terminal domain (115). Since they lack a transmembrane domain, ADAMTS members do not function as integral membrane proteins. The most relevant isoform for platelet activation, ADAMTS13, contains seven thrombospondin domains and two CUB domains; it is secreted by liver cells into the circulation (115).

The proteolytic activity of ADAMTS13 affects platelet function in an indirect way, as it cleaves ultralarge multimers of VWF (residues Tyr^1605^ and Met^1606^), which are secreted by the endothelium (1). This cleavage weakens the interaction of VWF with platelets. For ADAMTS13 to become enzymatically active, vascular VWF multimers need to change into an elongated form, such as is triggered by high shear conditions (116). By implication, at sites of blood stasis, the ADAMTS13 activity is low. By shear-dependent cleavage of VWF multimers, ADAMTS13 has an important role in the hemostatic regulation. Thus, in patients with low ADAMTS13 activity, platelets become more easily activated by the ultralarge VWF multimers, which can lead to a condition of thrombotic thrombocytopenic purpura (TTP) (117). In patients with a congenital form of TTP (Upshaw–Schulman syndrome), recessive mutations of ADAMTS13 are present, while in patients with acquired TTP, autoantibodies are observed targeting ADAMTS13 (118). In addition, low ADAMTS13 activity has been associated with arterial thrombotic conditions such as stroke (119).

## Intracellular Calpains and Caspases

The calpain isoforms mu, 1 and 2, act as cytosolic proteases, which rely for their enzymatic activity on rises in Ca^2+^ (120). While studies in the early 1990s pointed to a role of calpains in (thrombin-induced) shape change, linked to actin cytoskeletal rearrangements (121), it later became clear that calpains regulate multiple signaling proteins including integrin α_IIb_β_3_ (122–124). Calpains in particular appeared to play a role under conditions of prolonged rises in Ca^2+^ and contribute to agonist- or complement-induced shedding of microparticles (extracellular vesicles) (125, 126).

**Figure 4:**
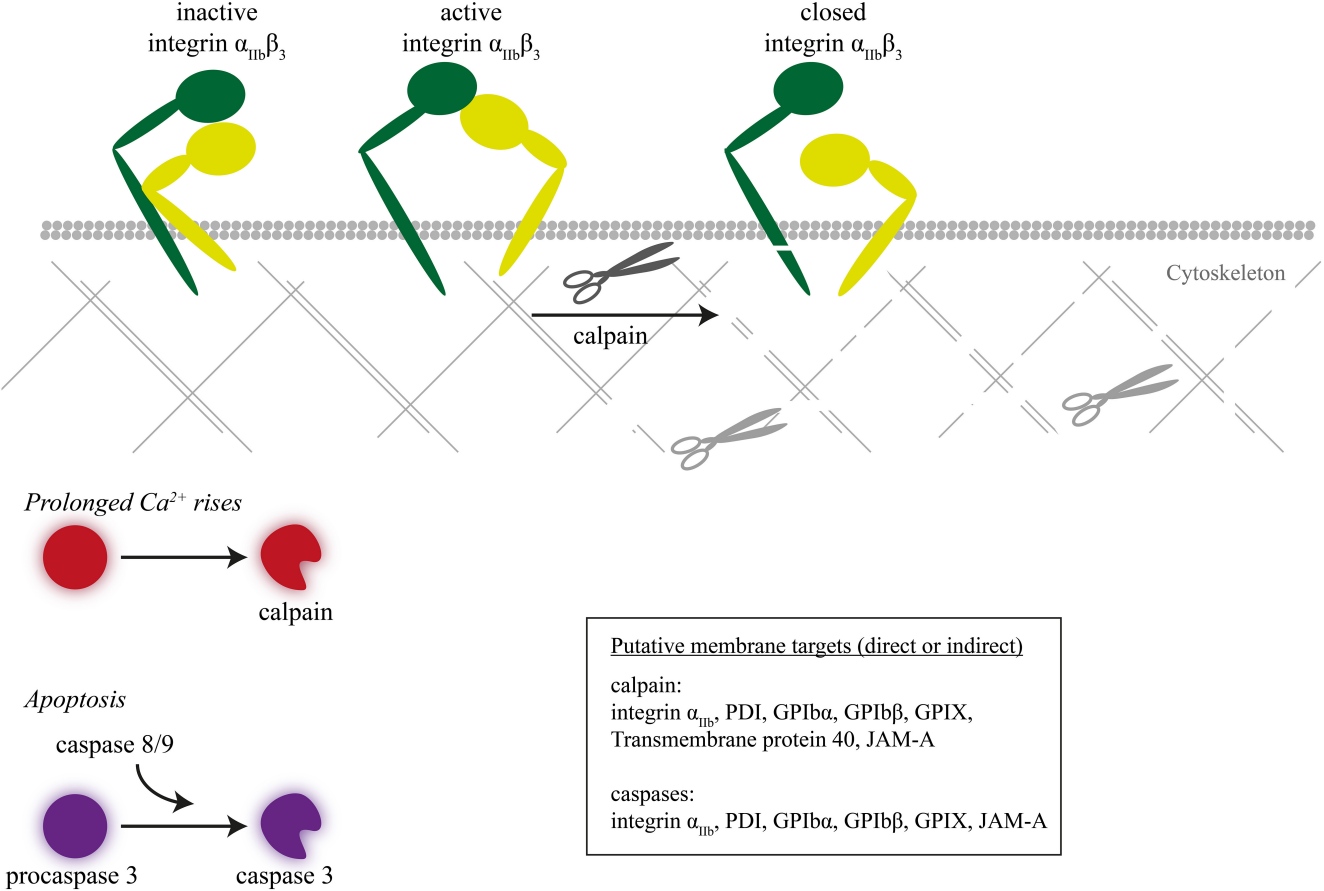
Inactivation of integrin β_3_ chain by intracellular proteolysis. Intracellular proteolysis mediated by calpains upon prolonged Ca^2+^ rise or by caspases during apoptosis leads to cleavage of the integrin β_3_ chain, next to cytoskeletal-linked signal proteins.

Regarding calpain receptor substrates in platelets, a marked finding was that calpains under certain, high-Ca^2+^ conditions can cleave the intracellular domain of the integrin β_3_ chain (Figure 4) (127). Markedly, this proteolysis was confined to β_3_ integrins of procoagulant, phosphatidylserine-exposing platelets (5). A recent proteomic neo N-terminal analysis of highly activated platelets (thrombin/convulxin or ionomycin) revealed up to 180 neo-N-terminal peptides sensitive to calpain inhibition, which corresponded to 106 proteins in particular cytoskeletal (filamin-A, talin-1, kindlin-3) and signaling proteins (128). Receptors cleaved by calpain included integrin β_3_, α_IIb_, and GPIbα, GPIbβ, GPIX, and CD226. Exactly which calpain isoform cleaves which intracellular proteins is still unclear.

Caspases (cysteine-aspartic proteases) are a family of proteolytic enzymes controlling programmed cell death or apoptosis. Presently, 12 caspase isoforms are known in man, which carry out the degradation of intracellular proteins without causing tissue necrosis. Caspases, once activated by proapoptotic stimuli, selectively cleave target proteins after an aspartic acid residue. In apoptotic platelets, the caspase pathways are also associated with phosphatidylserine exposure (129). When applied to platelets, the caspase-activating compound ABT-737 (an inhibitor of mitochondrial Bcl-2/Bcl-xL) appeared to act largely Ca^2+^ independently, implying caspase activities operating apart from calpains (130). In apoptotic platelets, especially the caspase-9 isoform plays a key role (131). Proteomic neo N-terminal analysis of ABT-737 stimulated platelets revealed 23 peptides and proteins (receptor, cytoskeletal, and signaling) sensitive to caspase inhibition, all with cleavage sites distinct from those of calpains (128). Next to integrin-associated proteins (filamin-A, kindlin-3), these were the integrin β_3_ and α_IIb_ chains, GPIbα and GPIbβ, i.e., cleavage sites compatible with a defective adhesive phenotype.

## Receptor Cleavage in Distinct Platelet Populations

In general, for the majority of proteases discussed, the proteolytic activity toward platelets increases upon activation. Platelets simultaneously exposed to collagen and thrombin or to a Ca^2+^ ionophore are characterized by prolonged cytosolic Ca^2+^ rises and ensuing exposure of phosphatidylserine (132). It is generally recognized now that these procoagulant platelets form a distinct population, morphologically characterized by swelling, ballooning, and microvesiculation (11, 133). Both phosphatidylserine exposure and ballooning are absent in activated platelets from Scott syndrome patients, lacking the Ca^2+^-activated channel protein anoctamin-6 (gene *ANO6* or *TMEM16F*), as well as in activated platelets from *Ano6* deficient mice (134).

Emerging evidence indicates that the high-Ca^2+^, phosphatidylserine-exposing platelets are focal sites of high protease activities on receptors and other membrane-bound proteins. For instance, a high activity of MMPs and ADAMs has been reported on platelets stimulated with collagen plus thrombin (73, 110). This type of stimulation appeared to increase the ADAM-mediated shedding of GPIbα and GPVI by 2.5-fold in comparison to single agonist stimulation (110). Markedly, shedding occurred normally in platelets from Scott patients, indicating that phosphatidylserine exposure is not a prerequisite for ADAM activity. As another example, the high Ca^2+^ rises in the phosphatidylserine-exposing platelet population potently stimulated intracellular receptor and actin cytoskeletal proteolysis by calpains (128). In addition, the coagulation-related proteolytic events are linked to platelet phosphatidylserine exposure. Accordingly, these platelets greatly promote coagulation factor proteolysis via tenase (factor Xa) and prothrombinase (thrombin) complexes (11). Herein, the binding of coagulation factors and the formation of fibrin can be further enhanced by ADAM-mediated glycoprotein shedding (110). In hemophilic (bleeding) conditions, these proteolytic events are greatly lowered (1). Along the same line, anticoagulant drugs suppress these platelet proteolytic activities, via inhibition of both coagulation factor proteolysis and metalloproteinase-mediated proteolysis (49, 135). In addition, phosphatidylserine-exposing platelets concentrate the protease plasmin, which stimulates platelet-dependent fibrinolysis (60).

A similar concentration of proteolytic events may occur in platelets that undergo apoptosis, e.g., induced by the drug ABT-737. Apoptotic platelets undergo caspase-dependent proteolytic events and phosphatidylserine exposure (130), although their procoagulant activity may be limited (129, 136). An apoptosis-like process is seen in (cold) stored platelets. Herein, sheddases like ADAM17 can induce the extracellular cleavage of GPIbα, GPV, and GPVI chains (137, 138), in a way that a lower GPVI expression correlated with a poorer platelet responsiveness (139). By storage with protease inhibitors or in mouse platelets lacking ADAM17, the hemostatic function of transfused platelets was improved (98, 140, 141). These findings provide novel options to improve platelet responsiveness upon transfusion.

## Concluding Remarks

Conclusively, on platelets, a range of proteases from various families—coagulation-related proteases, plasmin, MMPs, ADAM(TS) isoforms, cathepsins, caspases and calpains—appear to selectively activate or inactivate key adhesive and signaling receptors, thereby controlling platelet responses and functions. Furthermore, the high receptor-targeted proteolytic activity on phosphatidylserine-exposing (procoagulant) platelets implies a downregulation of the fractions of adhesive and aggregating platelets. Although relevant knowledge is gained on the regulation of these platelet- and plasma-derived proteases and their implications for platelet activation, still a better understanding of the spatiotemporal proteolytic activity within and in between platelets is needed to fully understand the complexity of receptor proteolysis upon thrombus formation in thrombosis and hemostasis.

## Author Contributions

JW, JH, and CB performed a literature search and wrote and revised the manuscript. All authors have seen and approved the final manuscript.

## Conflict of Interest

JH is a shareholder and cofounder of FlowChamber. The remaining authors declare that the research was conducted in the absence of any commercial or financial relationships that could be construed as a potential conflict of interest.
